# Food and water insecurity in households of children and adolescents living with HIV and receiving care in a rural Zambian hospital: A mixed-methods study

**DOI:** 10.1371/journal.pone.0300033

**Published:** 2024-06-04

**Authors:** Amanda C. Palmer, Phillimon Ndubani, Molly Sauer, Kathryn L. Spielman, Francis Hamangaba, Nkumbula Moyo, Bornface Munsanje, William J. Moss, Catherine G. Sutcliffe

**Affiliations:** 1 Department of International Health, Johns Hopkins Bloomberg School of Public Health, Baltimore, Maryland, United States of America; 2 Macha Research Trust, Choma, Zambia; 3 Department of Epidemiology, Johns Hopkins Bloomberg School of Public Health, Baltimore, Maryland, United States of America; Federal University of Agriculture Abeokuta, NIGERIA

## Abstract

Approximately 62,000 Zambian children are living with HIV. HIV care and treatment is generally more limited in rural areas, where a heavy reliance on rain-fed subsistence agriculture also places households at risk of food and water insecurity. We nested a mixed methods study with an explanatory sequential design in a clinical cohort of children and adolescents living with HIV (CHIV) in rural Zambia. We used validated questionnaires to assess household food and water insecurity and examined associations between indicators derived from those scales, household characteristics, and HIV treatment adherence and outcomes using log-binomial regression. We identified caregivers and older CHIV from food insecure households for in-depth interviews. Of 186 participants completing assessments, 72% lived in moderately or severely food insecure households and 2% in water insecure households. Food insecurity was more prevalent in households of lower socioeconomic status (80% vs. 59% for higher scores; p = 0.02) and where caregivers had completed primary (79%) vs. secondary school or higher (62%; p = 0.01). No other characteristics or outcomes were associated with food insecurity. Parents limited both the quality and quantity of foods they consumed to ensure food availability for their CHIV. Coping strategies included taking on piecework or gathering wild foods; livestock ownership was a potential buffer. Accessing sufficient clean water was less of a concern. During periods of drought or service interruption, participants travelled further for drinking water and accessed water for other purposes from alternative sources or reduced water use. Community contributions afforded some protection against service interruptions. Overall, while food insecurity was prevalent, strategies used by parents may have protected children from a measurable impact on HIV care or treatment outcomes. Reinforcing social protection programs by integrating livestock ownership and strengthening water infrastructure may further protect CHIV in the case of more extreme food or water system shocks.

## Introduction

Of the estimated 38 million people living with HIV worldwide, 67% live in Sub-Saharan Africa (SSA) [1]. SSA also accounts for almost 90% of the 1.7 million children and adolescents aged <15 years living with HIV globally [2], with the greatest burden concentrated in eastern and southern African countries. This region also faces the persistent threat of food insecurity triggered by rainfall shortages and crop failures [3]. Rural populations are of heightened concern on both fronts [4]. They have more limited access to HIV care and treatment [5] and, given a heavy reliance on rain-fed subsistence agriculture, experience wide fluctuations in the availability of, access to, and utilization of both food and water [4].

Food insecurity is thought to affect HIV treatment and care through either a mental health / behavioral pathway, i.e., influencing care-seeking and treatment adherence, or via a nutritional pathway, whereby inadequate dietary intakes lead to poor nutritional status and compromised immunity [6]. Poor access to adequate and safe water can similarly affect behavior (e.g., increasing caregiver burden) or can have implications for hygiene practices, which may increase the risk of opportunistic infections [7]. In the case of both food and water insecurity, most evidence has come from studies of adults living with HIV [8, 9]. The implications of these conditions for the continuity of care and treatment outcomes in children and adolescents likely differ. The literature is also more heavily weighted towards urban or peri-urban settings [10, 11], where the risk of food and water insecurity and any associated coping mechanisms are likely to differ from more rural communities [12]. It is important to understand the nature of these relationships in different settings to inform monitoring and prevention efforts.

We took advantage of an ongoing clinical cohort study of children and adolescents living with HIV in an area of rural Zambia where there are limitations in access to care and a reliance on agricultural practices prone to food and water system shocks. Specifically, our aims were to describe the prevalence of food and water insecurity in this cohort and to examine associations between food or water insecurity and HIV treatment adherence or outcomes. Using qualitative methods, we then sought to identify coping mechanisms and entry points to mitigate the impact of stressors in households with children and adolescents living with HIV.

## Methodology

### Study setting

This study was conducted at Macha Hospital in Choma District, Southern Province, Zambia. Macha Hospital is a 208-bed district-level referral hospital located approximately 70 km from the nearest town of Choma. It serves a population of over 150,000 persons, consisting mainly of subsistence farmers living in small, scattered homesteads, characteristic of much of rural Sub-Saharan Africa. The HIV prevalence in Southern Province was estimated to be 12.5% in 2016 [13]. Since 2005, Macha Hospital has operated an HIV clinic, providing HIV care and treatment services through the Government of Zambia’s antiretroviral treatment program, with additional support from the President’s Emergency Plan for AIDS Relief (PEPFAR). HIV care and treatment are provided free of charge by clinicians and clinical officers and adherence counseling by trained counselors.

We are unaware of any prior studies assessing food or water insecurity using household- or individual-levels using questionnaires in Choma District. However, parts of Southern Province are regularly classified as stressed based on monitoring of climate data, crop production, and agricultural pest outbreaks [14]. The province was also affected by a drought in 2019 [15].

### Study design

Our mixed methods study with an explanatory sequential design was nested within the Pediatric Antiretroviral Treatment (PART) study [16, 17]. PART is was prospective open clinical cohort study that started in September 2007. It was designed to assess the response to therapy and survival of children and adolescentsliving with HIV and receiving ART, as well as to monitor changes over time in the profile of children diagnosed with HIV and seeking care as national HIV guidelines and services evolved. Children and adolescents younger than 16 years of age living with HIV and receiving care at the HIV clinic were eligible for enrollment into the cohort. Child and household characteristics were assessed at the time of enrollment, including the collection of information on demographic and household characteristics and medical history, and then participants were evaluated at their quarterly clinic visits. At each visit, data were collected on treatment regimens, pills counts were recorded for all the participant’s medications, and weight and height measurements were taken. Results of routine laboratory tests, including CD4+ T-cell count and percentage and HIV viral load, were abstracted from medical records.

For the present mixed methods study, we initially considered all active (attending a study visit during the study period) participants in the PART cohort in March 2021 to be eligible for the cross-sectional component. We added assessments of food and water insecurity to the study questionnaire, which involved the use of two perception-based questionnaires. The Household Food Insecurity Access Scale (HFIAS) is a nine-item scale capturing data regarding three domains of food insecurity (1. anxiety about the household food supply; 2. insufficient quality of food; and 3. insufficient food intake and its consequences) and their frequency of occurrence (never, rarely, sometimes, or often) over the prior month [18]. The Household Water InSecurity Experiences (HWISE) Scale includes 12 items designed to assess household water access, availability, and use [19], with responses capturing the frequency of occurrence over the prior month (never, rarely, sometimes, often, or always).

For the qualitative component of this mixed methods study, we aimed to describe experiences of food or water insecurity in households with children and adolescents living with HIV and identify potential entry points for mitigating the impact of food- or water-related stressors. Participants were identified from the cross-sectional study through purposive sampling from among those classified as moderately or severely food insecure with the highest water insecurity scores, based on the scales and cut-offs described. We conducted 32 in-depth interviews with adolescents living with HIV (n = 16) and parents or caregivers of children living with HIV (n = 16). Trained data collectors conducted semi-structured in-depth interviews in Chitonga from June to October 2021. These were audio-recorded, transcribed, and translated into English. Study team members validated each transcript to correct any errors in transcription or translation.

### Statistical analysis

#### Quantitative data

The analysis included 182 (74%) of the 246 participants active in the PART cohort between March 2021 and February 2022 (10 excluded for being at boarding school, 5 declined, and 49 did not have a caregiver present at the visit to provide consent). We calculated a continuous HFIAS score, with a potential range from zero to 27, by summing the frequency of occurrence (never = 0, rarely = 1, sometimes = 2, or often = 3) across each of the nine food insecurity-related items. We then generated a categorical Food Insecurity Status indicator based on the frequency of experiences within each of the three HFIAS domains, as previously described [18]. Briefly, households were classified as food secure if they never or rarely worried about food (domain 1) but made no changes in food quality or intake (domains 2 or 3). The mild food insecurity category included households that sometimes or often worried about food (domain 1), consumed less preferred foods (domain 2), or either rarely or sometimes consumed a more limited variety or unwanted foods (domain 2). The moderate food insecurity category included households that sometimes or often consumed a more limited variety or unwanted foods (domain 2), or rarely/sometimes reduced the amount of food consumed (domain 3). The severe food insecurity category included households that often reduced the amount of food consumed or experienced having no food to eat, going to sleep hungry, or going the whole day and night without eating anything (domain 3). The HWISE score was calculated by summing the frequency of occurrence (never = 0, rarely = 1, sometimes = 2, often/always = 3) across each of the 12 items, for a score that could range from zero to 36 [19]. A standard cut-off of ≥12 was used to indicate a water-insecure household. The proportion of participants within each category of food and water insecurity was summarized overall and by season, with seasons defined as wet/rainy (mid-November to April), cool/dry (May to mid-August), and hot/dry (mid-August to mid-November). As the prevalence of water insecurity was low, we included only food insecurity in further analyses.

We summarized sociodemographic characteristics using data collected at the time of the participant’s enrollment into the cohort, which took place at a median of 10.2 years (25^th^, 75^th^ percentiles: 6.9, 12.0) prior to the administration of the HFIAS and HWISE scales. We calculated a socioeconomic status scale using data on housing characteristics, drinking water source, energy used for cooking, and asset ownership, as previously described [20], and grouped households into quartiles corresponding to scores of 0–6 (quartile 1; lowest), 7–12 (quartile 2), 13–18 (quartile 3), and 19–24 (quartile 4; highest). We summarized clinical characteristics from data collected at the same study visit as the administration of the HFIAS and HWISE scales. For children <5 years of age, we calculated weight-for-age Z scores (WAZ) using sex-specific standards [21] and classified children as underweight based on WAZ <-2. For older children and adolescents, we calculated body mass index (BMI) by dividing the child’s weight (kg) by his or her height squared (m^2^), derived BMI-for-age Z scores (BAZ) using sex-specific reference data [22], and classified children as thin based on BAZ <-2. Adherence was defined based on the lowest pill count recorded across all medications prescribed to the participant. We used a cut-off of ≤95% of pills to indicate non-adherence. If data from routine laboratory tests were missing from the HFIAS/HWISE visit, we used the closest measurement of CD4+ T-cell count or HIV viral load taken within 12 months prior to the study visit. We used a cut-off of <500 cells/mm^3^ to define a low CD4+T-cell count. Cut-offs of <50 copies/mL and <1000 copies/mL were used to define undetectable viral load and viral suppression, respectively.

The characteristics of study participants were summarized overall and by category of the household Food Insecurity Status indicator. Comparisons across groups were made using a chi-square test for categorical variables and Kruskal-Wallis test for continuous variables. As participants with none/mild and moderate/severe food insecurity were similar (S1 Table in S1 File), these groups were combined for the final analysis. Correlates of moderate/severe food insecurity, including sociodemographic characteristics and markers of nutritional status and treatment response, were explored using log-binomial regression to estimate prevalence ratios and 95% confidence intervals (CI). Sociodemographic characteristics that were marginally (p<0.2) correlated with food insecurity along with the marker of nutritional status or treatment response that was most strongly correlated with food insecurity in univariable analysis were considered for inclusion in a multivariable model (S2 Table in S1 File).

All analysis was conducted using Stata Version 17 (StataCorp, College Station, Texas). A p-value of <0.05 was considered statistically significant.

#### Qualitative data

Two study team members analyzed interview transcripts to inductively identify emergent themes. The two study team members conducted two rounds of open coding to develop and refine a code list. The final code list was then applied to all 32 transcripts, using ATLAS.ti Web Version 4.8.0-2023-01-14 (ATLAS.ti Scientific Software Development GmbH, Berlin, Germany) for analysis and data management. All transcripts were coded by at least one study team member; a randomly selected subset of 25% of transcripts were coded by two study team members. One study team member calculated inter-rater reliability with an additional randomly selected subset of 4 transcripts (12%). Reliability was >92%. The study team then discussed themes and patterns emerging from the analysis.

### Ethics statement

This study was approved by the Institutional Review Board at the Johns Hopkins Bloomberg School of Public Health, the University of Zambia Biomedical Research Ethics Committee, and the National Health Research Authority in Zambia. Adult participants provided written informed consent and caregivers of child participants provided parental permission. Participants 8–15 years of age provided assent.

## Results

### Cross-sectional study of food and water insecurity

**Fig 1:**
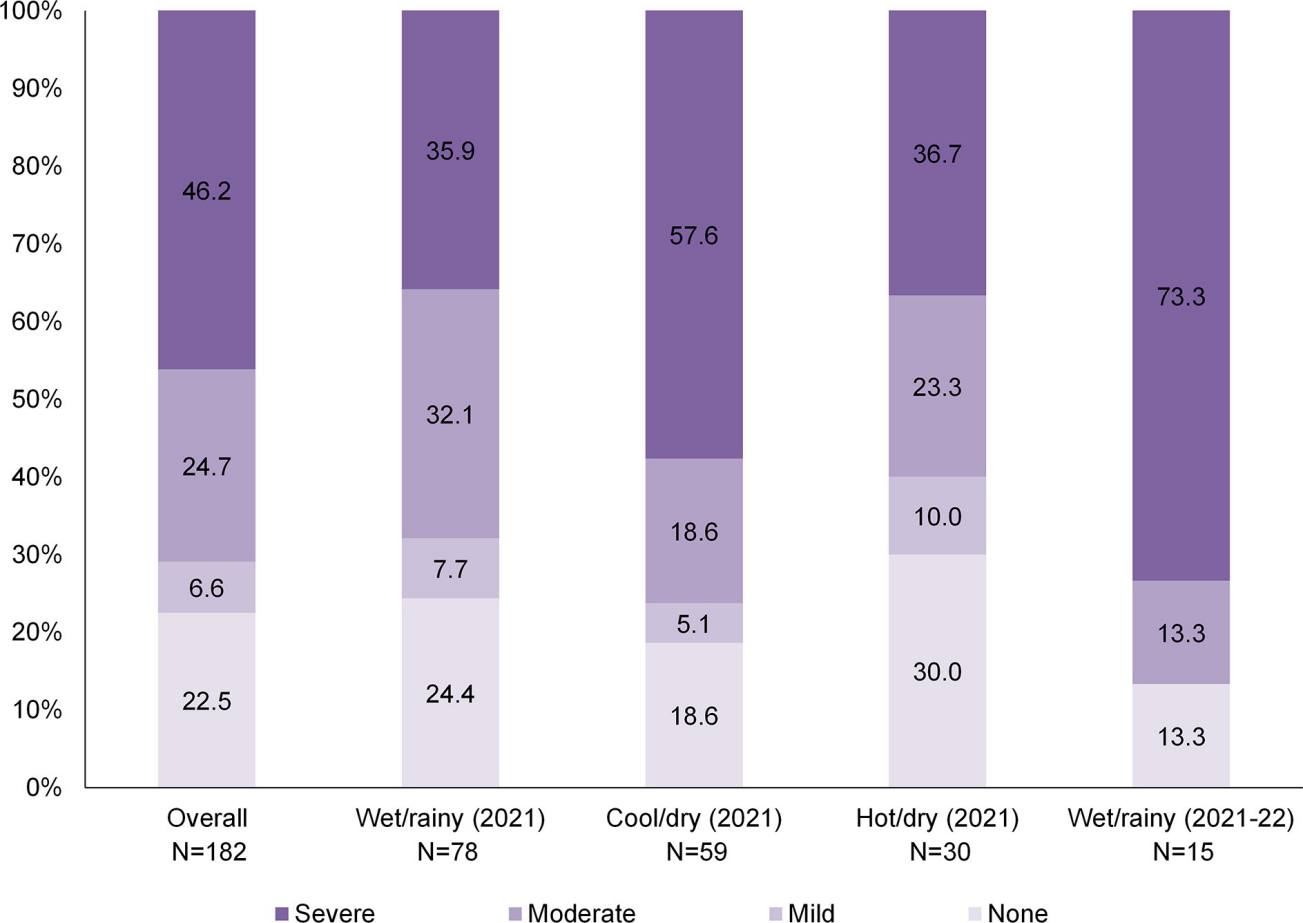
Household food insecurity reported by caregivers of children and adolescents living with HIV and receiving care in a rural hospital in Macha, Zambia from March 2021 to February 2022. Food insecurity was measured using the Household Food Insecurity Access Scale (HFIAS) [18] and categorized according to guidelines. Seasons were defined as wet/rainy (mid-November to April), cool/dry (May to mid-August), and hot/dry (mid-August to mid-November).

A total of 182 participants (49.5% female and a median age of 13.7 years) completed the HFIAS and HWISE Scales during the one-year study period (Table 1). The median score on the HFIAS scale was 5 (25^th^, 75^th^ percentiles: 1, 7; range: 0, 16). Based on the categorical Food Insecurity Status indicator, most households were classified as moderately (24.7%) or severely (46.2%) food insecure, with no significant differences by season (p = 0.14; Fig 1). The median score on the HWISE scale was 0 (25^th^, 75^th^ percentiles: 0, 2; range: 0, 16). Only 3 participants (1.7%) were classified as living in a water insecure household, including 1 (33.3%) who also had moderate food insecurity and 2 (66.7%) who also had severe food insecurity (exact p = 1.00 for comparison of water insecurity by category of food insecurity).

**Table 1:** Characteristics of households and children and adolescents living with HIV and receiving care in a rural hospital in Macha, Zambia, overall and by level of food insecurity.

	Overall (n = 182)	Food secure or mildly food insecure (n = 53)	Moderately or severely food insecure (n = 129)	p-value
**Demographic characteristics**				
Socioeconomic status, n (%) ^a^^,^^b^				0.007
Quartile 1 (lowest)	103 (57.2)	21 (40.4)	82 (64.1)	
Quartile 2	59 (32.8)	23 (44.2)	36 (28.1)	
Quartile 3	16 (8.9)	6 (11.5)	10 (7.8)	
Quartile 4 (highest)	2 (1.1)	2 (3.9)	0	
Caregiver’s highest level of education, n (%) ^a^				0.01
None/primary	103 (71.4)	23 (44.2)	80 (62.5)	
Secondary	50 (27.8)	15 (28.9)	35 (27.3)	
College/Certificate	27 (15.0)	14 (26.9)	13 (10.2)	
Age in years, median (25^th^, 75^th^ percentiles)	13.7 (10.5, 17.7)	15.5 (11.1, 19.1)	13.4 (10.1 16.6)	0.17
Sex, n (%)				0.80
Male	92 (50.5)	26 (49.1)	66 (51.2)	
Female	90 (49.5)	27 (50.9)	63 (48.8)	
Parent’s vital status, n (%)				0.49
Both alive	130 (71.4)	35 (66.0)	95 (73.6)	
One parent died	40 (22.0)	13 (24.)	27 (20.9)	
Both died	12 (6.6)	5 (9.4)	7 (5.4)	
**Nutritional, immunologic, virologic and treatment characteristics**				
Underweight / thin, n (%) ^c^	21 (14.3)	4 (10.0)	17 (15.9)	0.36
Time on ART in years, median (25^th^, 75^th^ percentiles)	9.0 (6.8, 11.5)	9.7 (6.9, 12.1)	8.8 (6.8, 11.3)	0.27
CD4+ T-cell count in cells/mm^3^, median (25^th^, 75^th^ percentiles) ^d^	803 (615, 1078)	736 (548, 1060)	818 (622, 1093)	0.57
CD4+ T-cell <500 cells/mm^3^, n (%) ^d^	17 (14.7)	6 (18.8)	11 (13.1)	0.44
HIV viral load <50 copies/mL, n (%) ^e^	118 (80.3)	37 (84.1)	81 (78.6)	0.45
HIV viral load <1000 copies/mL, n (%) ^e^	138 (93.9)	42 (95.5)	96 (93.2)	0.60
Adherence ≤95%, n (%) ^f^	35 (27.8)	10 (24.4)	25 (29.4)	0.56
ART regimen, n (%)				0.29
ABC/3TC/DTG	20 (11.0)	4 (7.6)	16 (12.4)	
ABC/3TC/EFV	1 (0.6)	0	1 (0.8)	
ABC/3TC/LPVR	18 (9.9)	8 (15.1)	10 (7.8)	
AZT/3TC/DTG	2 (1.1)	1 (1.9)	1 (0.8)	
AZT/3TC/LPVR	16 (8.8)	2 (3.8)	14 (10.9)	
TAF/FTC/DTG	34 (18.7)	9 (17.0)	25 (19.4)	
TDF/3TC/DTG	81 (44.5)	24 (45.3)	57 (44.2)	
TDF/3TC/EFV	1 (0.6)	1 (1.9)	0	
TDF/FTC/DTG	1 (0.6)	0	1 (0.8)	
TDF/FTC/EFV	7 (3.9)	4 (7.6)	3 (2.3)	
TDF/FTC/LPVR	1 (0.6)	0	1 (0.8)	

3TC: lamivudine; ABC: abacavir; ART: antiretroviral therapy; AZT: zidovudine; DTG: dolutegravir; EFV: efavirenz; FTC: emtricitabine; LPVR: lopinovir/ritonavir; TAF: tenofovir alafenamide; TDF: tenofovir disoproxil fumarate

^a^ Measured at enrollment into the cohort; all other characteristics measured at study visit where HFIAS and HWISE modules were administered [median = 10.2 years (25^th^, 75^th^ percentiles: 6.9, 12.0) after enrollment]

^b^ Based on socioeconomic status scale derived from data on housing characteristics, drinking water source, energy used for cooking, and asset ownership [20]; grouped into quartiles corresponding to scores of 0–6 (quartile 1; lowest), 7–12 (quartile 2), 13–18 (quartile 3), and 19–24 (quartile 4; highest)

^c^ Underweight defined by weight-for-age Z score <-2 for children <5 years of age; thin defined by body mass index-for-age Z score <-2 for children and adolescents ≥5 years of age

^d^ CD4 only available for 116 participants (63.7%); closest measure within 12 months prior the study visit was used [median = 182 days (25th, 75th percentiles: 0, 266) from measurement to study visit]

^e^ HIV viral load only available for 147 participants (80.8%); closest measure within 12 months prior the study visit was used [median = 84 days (25th, 75th percentiles: 0, 182) from measurement to study visit]

^f^ Adherence only available for 126 participants (69.2%); measured by pill count and defined based on the lowest measure for all drugs counted at the study visit

In univariable analysis, lower socioeconomic status and caregiver’s education were significantly correlated and younger age was marginally correlated with higher prevalence of moderate or severe food insecurity (Tables 1 and 2). Markers of nutritional status and treatment response were not significantly correlated with moderate or severe food insecurity. In a multivariable model, only lower socioeconomic status remained significantly correlated with higher prevalence of moderate or severe food insecurity (Table 2).

**Table 2:** Household- and child-level correlates of moderate or severe food insecurity in a cohort of children and adolescents living with HIV and receiving care in a rural hospital in Macha, Zambia.

	Total N	Moderate or severe food insecurity N (%)	Crude prevalence ratio (95% confidence interval)	Adjusted prevalence ratio (95% confidence interval)
Socioeconomic status ^a^^,^^b^				
Quartile 1 (lowest)	103	82 (79.6)	Ref	Ref
Quartile 2	59	36 (61.0)	0.77 (0.61, 0.96)	0.75 (0.58, 0.97)
Quartiles 3 & 4 (highest)	18	10 (55.6)	0.70 (0.46, 1.07)	0.84 (0.54, 1.33)
Caregiver’s highest level of education ^a^				
None/primary	103	80 (77.7)	Ref	Ref
Secondary	50	35 (70.0)	0.90 (0.73, 1.11)	1.00 (0.77, 1.30)
College/Certificate	27	13 (48.2)	0.62 (0.41, 0.93)	0.79 (0.51, 1.24)
Age (years)				
0–9	42	32 (76.2)	Ref	Ref
10–14	62	48 (77.4)	1.02 (0.82, 1.26)	0.98 (0.77, 1.25)
≥15	78	49 (62.8)	0.82 (0.65, 1.05)	0.75 (0.57, 1.00)
HIV viral load (copies/mL) ^c^				
≥50	29	22 (75.9)	1.11 (0.87, 1.40)	1.11 (0.88, 1.41)
<50	118	81 (68.6)	Ref	Ref

^a^ Measured at enrollment into the cohort; all other characteristics measured at study visit where HFIAS and HWISE modules were administered [median = 10.2 years (25th, 75th percentiles: 6.9, 12.0) after enrollment]

^b^ Based on socioeconomic status scale derived from data on housing characteristics, drinking water source, energy used for cooking, and asset ownership [20]; grouped into quartiles corresponding to scores of 0–6 (quartile 1; lowest), 7–12 (quartile 2), 13–18 (quartile 3), and 19–24 (quartile 4; highest)

^c^ HIV viral load only available for 147 participants (80.8%); closest measure within 12 months prior the study visit was used [median = 84 days (25th, 75th percentiles: 0, 182) from measurement to study visit]

### Qualitative study of food and water insecurity

Several key themes emerged from the semi-structured in-depth interviews of caregivers and adolescents with HIV and their experiences with food and water insecurity, including (1) coping with food insecurity, (2) seeking help from community, (3) reliability of water sources, and (4) experiences of children with HIV.

#### Coping with food insecurity

Adapting food sources and eating behaviors was frequently required by households, with caregivers generally more attuned to these shifts than adolescents. While *nshima* (stiff maize porridge) remained a staple, households changed the accompanying foods, sought them through gathering rather than gardening, or reduced portion sizes or eliminated meals altogether. Many described purchasing or seeking out maize from support networks to ensure *nshima* would be available.

Several caregivers detailed their reliance on piece work—short term task-based employment, such as gardening or making charcoal, for neighbors or others in the community in exchange for maize or money to purchase food—as they navigated both specific times of food insecurity and general challenges related to household economic status.

*“A piece work like maybe someone like these farmers planted a lot of maize*, *so when it’s time to harvest I join to do the work so when I stop working when the harvesting is done*, *they give me this*, *they give me maize for me to be eating at home*.*” (Caregiver)*

*"We were living by doing piece works for those who had*, *or you go and borrow to those that had just like that*, *or from the same profit of the business you were doing you take it to buy food*. *When you buy*, *if it’s a gallon [of maize] then you economize that’s how we were living we were struggling*, *we used to go and ask for help*, *you go to your relative you ask for help*, *they cannot refuse to help you*.*” (Caregiver)*

Livestock provided some protection from prolonged insecurity, as households were able to sell cattle, goats, or chickens to purchase maize or other foods. However, selling livestock as a means of coping with food insecurity was not always sufficient. The urgency and severity of food insecurity at times led to insufficient economic benefit from livestock sales and cascading psychosocial effects.

*“I work hard so that I find food for the children to eat*. *As I was saying that goats saved me just like that*, *you sell and you buy a bag of mealie meal*, *they eat*.*” (Caregiver)*

*“Ah…the goodness was that goats, cattle, and chickens helped us a lot. Because if we did not have these, we would have died. So we saw that it is good to keep chickens, goats and others so that they help when you are in need.” (Caregiver)*

*“During the difficult times I had problems of not having money to buy food for my family*, *until I started selling cattle at unacceptable low price*. *Lower price than the normal market price and that troubled me a lot*. *At some point we adults didn’t manage to eat nshima for 2 days apart from giving the children*, *we were just eating beans*. *This happened to me as a head of household*. *So it proved to me that I was really in a difficult situation*, *which should be not the case that we fail to eat nshima*, *because we are Tonga people*.*” (Caregiver)*

#### Seeking community help

Respondents described their reliance on relatives for help with buying or acquiring food, including both family members living locally and those outside the immediate community. Experiences and perceptions about seeking help from non-family community members varied among participants; some indicated norms around providing help if asked, while others indicated an expectation that households or individuals would need to help themselves and could not count on support from others.

*“Yes*, *just our neighbor who understands people’s problems*, *as long as you go to ask*, *it seems like she is rich*. *When you go to ask*, *she understands if you don’t have food*. *She will ask you to help her too*, *since she is old and she will tell you where to work from politely*, *so you can get food*.*” (Caregiver)*

*“…maybe I can just say that we differ in that maybe they have someone who lives in town who is maybe working. During difficult times he/she can send them money to use, but in short, we all have difficulties in the community we come from.” (Adolescent)*

*“…if the whole village is faced with this same problem, it is upon you as the owner of the home to work out a plan on how to survive.” (Caregiver)*

*“Yes*, *I didn’t feel good*, *I felt shy and I started having some doubt about asking*, *it’s only that I see how we help each other*.*” (Caregiver)*

#### Reliable water sources

Most respondents indicated relatively reliable access to water perceived to be safe for drinking, cooking, bathing, washing items, and watering livestock. The physical distance and time required to collect water for the household varied widely from just a few minutes to one hour. Boreholes were commonly noted as the primary source of water for drinking and cooking. Many water sources were dependent on community contributions to maintain and repair pumps and piping, helping to minimize disruptions to potable water access in some cases.

*“Every year you talk and agree that each family will give such an amount*. *Like last year*, *we had agreed that each family give K100*. *That K100 is meant for repairs when it breaks down*, *so that those who are in forefront [meaning organizers] can buy the damaged parts so that it can be fixed*.*” (Caregiver)*

In the event of water shortages or depleted or damaged local sources, respondents described seeking alternate sources for other uses (e.g., bathing, washing clothes). For example, when nearby boreholes were either broken or crowded, participants instead gathered water from shallow wells, streams, or other sources, or traveled to more distant boreholes.

*“At Sibanze where we used to fetch the hand pump broke down*, *now maybe hospital [meaning clinic] hand pump it is where we fetch*, *it is where we get*. *… and sometimes it also breaks down and you go to the stream and fetch*. *… Always we fetch from there [Sibanze]*, *it’s only that now it is broken down*, *because it is three months now since it broke down that’s why we started going this side*.*” (Caregiver)*

*“Water for drinking*, *the water we bring from shallow wells we just use it for other things*, *we go to the other borehole where we fetch water for drinking*. *We don’t drink water we fetch from shallow wells even though the other borehole is far maybe 2*.*5 kilometers where we fetch two 20-liter containers*, *specifically for drinking*. *We don’t use it for anything else*.*” (Caregiver)*

They also described changes to water use. These included preserving water from some sources specifically for drinking and adjusting the sources used for cleaning and watering animals or reducing the quantities of water used.

*“We change on how we use water*, *if you used to bath a 10L you start bathing 5L of water because water is difficult*. *Even the livestock don’t drink water like how they are supposed to*.*” (Caregiver)*

Despite a majority of respondents indicating confidence in accessing sufficient water for the household on a consistent basis, even in periods of lower rainfall, others described hardships finding safe, drinkable water from sources within reach.

*“There is no good in it*, *it’s bad that we lack hand pumps*. *Water is supposed to be nearby*. *It’s only that in our area there is no hand pump nearby*, *so we are suffering*.*” (Caregiver)*

*“There are no other ways*, *we live in hardship just like that*, *apart from going to the same boreholes even when they are far away because there is no other way*. *You can’t say I will drill here*. *I will do nothing*, *even if you go to the shallow wells*, *you will not find water*. *So apart from going to the same boreholes even when they are far away*, *so that you could have water to drink*, *because if you just sit*, *there is nowhere you will find water from*. *There is no other way*. *(Caregiver)*

#### Experiences of children with HIV

Ensuring adequate food and water for children and adolescents living with HIV during times of insecurity—whether acute, as in the 2019 drought, or seasonal—was a priority for caregivers. Many described concerns about taking antiretroviral therapy while hungry or without clean water and adapted their own behaviors to ensure the child living with HIV would have adequate food and water. Some adolescents living with HIV similarly highlighted treatment challenges when hungry, though less frequently than caregivers.

*“…the medicine the child is taking is not supposed to be taken when he is hungry*, *when there is no food in the body*, *he may grow thin*. *So it makes me to panic thinking that I will have problems with the child if I the parent don’t put more effort*.*” (Caregiver)*

*“Because of that drug*…*that food I am eating does not give me energy*, *it does not build my body which means I have to change so that I find other foods so that I become healthy*.*” (Adolescent)*

## Discussion

In this rural setting, we found a high prevalence of moderate or severe food insecurity, with ~70% of households affected. By contrast, fewer than 2% of households had experienced water insecurity. There was no relationship in our data between food insecurity and treatment adherence. Indicators of nutritional status and treatment response did not differ based on the food security status of the household. The in-depth interviews captured several of the responses to food-insecure periods, such as eating smaller portion sizes, that are known to be consistent across cultures [23] and several setting-specific coping strategies. As seen in the quantitative data, accessing sufficient clean water was less of a concern for most participants, although some did report the need to travel long distances. Participants reported that they altered water use during times of water shortage and when water sources were damaged.

The high prevalence of food insecurity and significant relationship between food insecurity and socioeconomic status were unsurprising. During this period, Choma District was classified as stressed due to a combination of the COVID-19 pandemic, elevated maize prices, and agricultural pest outbreaks [14]. The pandemic’s influence on food insecurity, in particular, has been well-described [24, 25]. Rural families relying on small-scale agriculture were thought to be particularly affected in Zambia, where the country’s policy responses to the pandemic affected social protection programs like fertilizer input supports [26]. As observed here, the poorest households are generally at greatest risk, with low wages considered among the major drivers of food insecurity [27]. Our qualitative interviews revealed anticipated changes in food quality (eating less preferred foods, e.g., beans instead of *nshima*) and quantity (smaller portion sizes, skilling meals). Participants reported various coping strategies such as taking on piecework or gathering wild foods. Livestock ownership was seen as a potential buffer. While many could rely on family members for assistance, it was not always considered acceptable to ask for help from others outside of the family.

Numerous observational studies in adults support a link between food insecurity and poor treatment adherence [28], including work carried out in Zambia [29]. More recent research has suggested that children may likewise be affected [30, 31]. In our study, we found no significant differences in antiretroviral therapy adherence among children in food insecure households. We know from qualitative research elsewhere in Africa that medications may be withheld from children when there is insufficient food due to a belief that the drugs are too strong to be taken on an empty stomach [32, 33]. In our in-depth interviews, parents raised concerns about their children taking antiretroviral therapy while hungry. However, they also reported altering their own food intake to ensure sufficient food for their child. This protective measure may have helped to maintain high adherence among children in our sample. Prior research has also shown that children may refuse to take medications when food is insufficient, as it helps to counteract side effects [34]. While this specific issue was not raised by adolescents in our study, some did note treatment challenges such as concerns that they needed to eat more to meet their nutritional needs.

We found no association in our data between food insecurity and HIV treatment outcomes. This is contrary to findings from two systematic reviews showing consistent relationships between food insecurity, lower CD4+ T cells counts [8], and lower odds of viral suppression [9]. It is important to note that these were largely based on studies in U.S. adults, with only one reporting on a cohort of adults in Uganda [35]. Research in children has been even more limited. We are aware of only one study, among 2-6-year-old outpatient children living with HIV in urban Botswana, which reported a significant association between CD4+ T cell counts and household food insecurity [11]. Effects on HIV treatment outcomes are thought to be partly attributable to lower adherence [6], which we did not observe in our data. Limited nutrient intakes and status could alternatively compromise immunity [6]. Again, however, our qualitative findings suggest that parents are protecting their children by reducing their own food intake. This would limit the negative effects through a nutritional pathway.

The low prevalence of water insecurity—apparent in both our quantitative and qualitative data—was unexpected, as this has been recognized as a major challenge and policy priority in Zambia [36, 37]. One study carried out in Lusaka found an almost 40% prevalence of water insecurity using the HWISE scale [38]. It is difficult to make a direct comparison here given differences in the timing of data collection and likely urban-rural differences in water resilience. While households may not have met the established HWISE cut-off, in-depth interviews did reveal challenges both during periods of water scarcity (e.g., during the 2019 drought) and service interruption (e.g., damaged pump). Consistent with prior research [39, 40], participants cited changes in water sourcing as one coping strategy: traveling further to access water, particularly for drinking, and gathering from shallow wells or streams for activities like cleaning or watering animals. Some also reported reducing water use, including for livestock. Community contributions to water infrastructure may have afforded some protection against service interruptions. The small number of water-insecure households in our cohort precluded analysis of its relationship with HIV treatment adherence and outcomes. For the former, at least one report from Zambia has shown a link between limited water access and adherence intention [41]. Research from multiple sites in SSA indicates that treatment adherence may be particularly affected during periods of drought [42], suggesting the need for continued monitoring of water insecurity at our research site. Water insecurity is an important driver of food insecurity. Thus, the combination of the two would likely have a more measurable impact on HIV outcomes.

Our study did have limitations. Participants in the ongoing clinical cohort in which we nested our study are not necessarily representative of all children living with HIV and receiving care in rural Zambia. It may be the case that food and water insecurity are more severe in households of children receiving care at more rural facilities, as opposed to a referral hospital, and that associations with HIV treatment adherence and outcomes may have been detectable under those circumstances. We were further limited by a cross-sectional design, with food and water insecurity and treatment adherence and outcomes measured at the same time, thus precluding an evaluation of the temporal association between measures. We were also bounded by a short time period. Food and water insecurity is likely to vary over time in this setting; further, there have been known periods of shock, such as during the 2019 drought. It is possible that any associations between food and water insecurity with adherence and treatment outcomes would have been more apparent in a longitudinal analysis or with an acute exposure. There were also limitations in some of our assessments. For example, in some instances we had to rely on laboratory data from an earlier timepoint. Even with these limitations, we were able to incorporate standardized assessments of food and water insecurity into data collection from a well-characterized cohort, enabling comparisons with reports from other settings. In addition, the sequential explanatory design helped to expand our understanding of the experiences of food and water insecurity and to identify some potential coping mechanisms that could be built on in future interventions.

## Conclusions

Our study provides critical baseline measures of food and water insecurity in a rural setting similar to many areas of SSA in terms of its vulnerability to rainfall shortages, crop failures, and livestock losses. Although food insecurity was prevalent, we found no association with HIV treatment adherence or outcomes. Such relationships are likely to become apparent with more severe shocks. As such, social protection programs should be reinforced, potentially expanding beyond current crop inputs to bolster livestock ownership. A successful example has recently been reported from Kenya [43], which notably reduced risky behaviors among adolescents [44]. We also found that households may be vulnerable to water insecurity during periods of low rainfall or broken equipment. It will be critical to continue monitoring both food and water insecurity in this setting going forward.

## Supporting information

S1 Checklist (DOC)

S1 File (DOCX)
