# Supplementary material for: Food and water insecurity in households of children and adolescents living with HIV and receiving care in a rural Zambian hospital: A mixed-methods study
Source: PLoS One. 2024 Jun 4;19(6):e0300033. doi: 10.1371/journal.pone.0300033 (PMC11149834; doi:10.1371/journal.pone.0300033)
Supplement: S1 File — (DOCX) [file pone.0300033.s002.docx]

**Supporting Information**

**Food and water insecurity in households of children and adolescents living with HIV and receiving care in a rural Zambian hospital: A mixed-methods study**

Amanda C. Palmer, Phillimon Ndubani, Molly Sauer, Kathryn L. Spielman, Francis Hamangaba, Nkumbula Moyo, Bornface Munsanje, William J. Moss, Catherine G. Sutcliffe

S1 Table. Characteristics of households and children living with HIV and receiving care in a rural hospital in Macha, Zambia, overall and by level of food insecurity

S2 Table. Household- and child-level correlates of moderate or severe food insecurity in a cohort of children living with HIV and receiving care in a rural hospital in Macha, Zambia

**S1 Table. Characteristics of households and children and adolescents living with HIV in Macha, Zambia, overall and by level of food insecurity**

|  | **Overall**  **(n=182)** | **None**  **(n=41)** | **Mild**  **(n=12)** | **Moderate**  **(n=45)** | **Severe**  **(n=84)** | **p-value** |
| --- | --- | --- | --- | --- | --- | --- |
| **Demographic characteristics** | | | | | | |
| Socioeconomic status, n (%) ^a,b^ |  |  |  |  |  | 0.09 |
| Quartile 1 | 103 (57.2) | 17 (42.5) | 4 (33.3) | 29 (64.4) | 53 (63.9) |  |
| Quartile 2 | 59 (32.8) | 17 (42.5) | 6 (50.0) | 13 (28.9) | 23 (27.7) |  |
| Quartile 3 | 16 (8.9) | 4 (10.0) | 2 (16.7) | 3 (6.7) | 7 (8.4) |  |
| Quartile 4 | 2 (1.1) | 2 (5.0) | 0 | 0 | 0 |  |
| Caregiver’s highest level of education, n (%) ^a^ |  |  |  |  |  | 0.10 |
| None/primary | 103 (71.4) | 18 (45.0) | 5 (41.7) | 31 (68.9) | 49 (59.0) |  |
| Secondary | 50 (27.8) | 11 (27.5) | 4 (33.3) | 10 (22.2) | 25 (30.1) |  |
| College/Certificate | 27 (15.0) | 11 (27.5) | 3 (25.0) | 4 (8.9) | 9 (10.8) |  |
| Age in years, median (25th, 75th percentiles) | 13.7 (10.5, 17.7) | 15.8 (11.6, 19.9) | 12.9 (10.7, 18.4) | 13.1 (10.1, 16.5) | 13.5 (10.3, 16.7) | 0.48 |
| Sex, n (%) |  |  |  |  |  | 0.82 |
| Male | 92 (40.5) | 21 (51.2) | 5 (41.7) | 25 (55.6) | 41 (48.8) |  |
| Female | 90 (49.5) | 20 (48.8) | 7 (58.3) | 20 (44.4) | 43 (51.2) |  |
| Parent’s vital status, n (%) |  |  |  |  |  | 0.90 |
| Both alive | 130 (71.4) | 27 (65.9) | 8 (66.7) | 31 (68.9) | 64 (76.2) |  |
| One parent died | 40 (22.0) | 10 (24.4) | 3 (25.0) | 11 (24.4) | 16 (19.1) |  |
| Both died | 12 (6.6) | 4 (9.8) | 1 (8.3) | 3 (6.7) | 4 (4.8) |  |
| **Nutritional, immunologic, virologic and treatment characteristics** | | | | | | |
| Underweight / thin, n (%) ^c^ | 21 (14.3) | 3 (10.0) | 1 (10.0) | 6 (16.2) | 11 (15.7) | 0.84 |
| Time on ART in years, median (25th, 75th percentiles) | 9.0 (6.8, 11.5) | 9.7 (6.8, 12.3) | 9.8 (7.0, 11.7) | 8.9 (7.4, 11.5) | 8.7 (6.4, 11.3) | 0.56 |
| CD4+ T-cell count in cells/mm^3^, median (25th, 75th percentiles)^d^ | 803 (615, 1078) | 765 (548, 1109) | 736 (587, 915) | 1013 (735, 1246) | 743 (590, 964) | 0.08 |
| CD4+ T-cell <500 cells/mm^3^, n (%) ^d^ | 17 (14.7) | 5 (20.8) | 1 (12.5) | 3 (9.7) | 8 (15.1) | 0.71 |
| HIV viral load <50 copies/mL, n (%) ^e^ | 118 (80.3) | 29 (82.9) | 8 (88.9) | 33 (89.2) | 48 (72.7) | 0.19 |
| HIV viral load <1000 copies/mL, n (%) ^e^ | 138 (93.9) | 34 (97.1) | 8 (88.9) | 36 (97.3) | 60 (90.9) | 0.42 |
| Adherence ≤95%, n (%) ^f^ | 35 (27.8) | 7 (21.9) | 3 (33.3) | 8 (25.8) | 17 (31.5) | 0.77 |
| ART regimen, n (%) |  |  |  |  |  | 0.81 |
| ABC/3TC/DTG | 20 (11.0) | 3 (7.3) | 1 (8.3) | 8 (17.8) | 8 (9.5) |  |
| ABC/3TC/EFV | 1 (0.6) | 0 | 0 | 0 | 1 (1.2) |  |
| ABC/3TC/LPVR | 18 (9.9) | 6 (14.6) | 2 (16.7) | 4 (8.9) | 6 (7.1) |  |
| AZT/3TC/DTG | 2 (1.1) | 1 (2.4) | 0 | 0 | 1 (1.2) |  |
| AZT/3TC/LPVR | 16 (8.8) | 2 (4.9) | 0 | 5 (11.1) | 9 (10.7) |  |
| TAF/FTC/DTG | 34 (18.7) | 6 (14.6) | 3 (25.0) | 7 (15.6) | 18 (21.4) |  |
| TDF/3TC/DTG | 81 (44.5) | 20 (48.8) | 4 (33.3) | 20 (44.4) | 37 (44.1) |  |
| TDF/3TC/EFV | 1 (0.6) | 1 (2.4) | 0 | 0 | 0 |  |
| TDF/FTC/DTG | 1 (0.6) | 0 | 0 | 0 | 1 (1.2) |  |
| TDF/FTC/EFV | 7 (3.9) | 2 (4.9) | 2 (16.7) | 1 (2.2) | 2 (2.4) |  |
| TDF/FTC/LPVR | 1 (0.6) | 0 | 0 | 0 | 1 (1.2) |  |
| 3TC: lamivudine; ABC: abacavir; ART: antiretroviral therapy; AZT: zidovudine; DTG: dolutegravir; EFV: efavirenz; FTC: emtricitabine; LPVR: lopinovir/ritonavir; TAF: tenofovir alafenamide; TDF: tenofovir disoproxil fumarate  ^a^ Measured at enrollment into the cohort; all other characteristics measured at study visit where HFIAS and HWISE modules were administered [median = 10.2 years (25th, 75th percentiles: 6.9, 12.0) after enrollment]  ^b^ Based on socioeconomic status scale derived from data on housing characteristics, drinking water source, energy used for cooking, and asset ownership [20]; grouped into quartiles corresponding to scores of 0-6 (quartile 1; lowest), 7-12 (quartile 2), 13-18 (quartile 3), and 19-24 (quartile 4; highest)  ^c^ Underweight defined by weight-for-age Z score <-2 for children <5 years of age; thin defined by body mass index-for-age Z score <-2 for children and adolescents ≥5 years of age  ^d^ CD4 only available for 116 participants (63.7%); closest measure within 12 months prior the study visit was used [median = 182 days (25th, 75th percentiles: 0, 266) from measurement to study visit]  ^e^ HIV viral load only available for 147 participants (80.8%); closest measure within 12 months prior the study visit was used [median = 84 days (25th, 75th percentiles: 0, 182) from measurement to study visit]  ^f^ Adherence only available for 126 participants (69.2%); measured by pill count and defined based on the lowest measure for all drugs counted at the study visit | | | | | | |

**S2 Table. Household- and child-level correlates of moderate or severe food insecurity** **in a cohort of children and adolescents living with HIV in Macha, Zambia**

|  | **Total N** | **Moderate/severe food insecurity**  **N (%)** | **Crude prevalence ratio (95% confidence interval)** | **Model 1: Adjusted prevalence ratio (95% confidence interval)** | **Model 2: Adjusted prevalence ratio (95% confidence interval)** | **Model 3: Adjusted prevalence ratio (95% confidence interval)** | **Model 4: Adjusted prevalence ratio (95% confidence interval)** | **Model 5: Adjusted prevalence ratio (95% confidence interval)** |  |
| --- | --- | --- | --- | --- | --- | --- | --- | --- | --- |
| **Demographic characteristics** | | | | | | | | | |
| Socioeconomic status ^a,b^ | | | | | | | | | |
| Quartile 1 | 103 | 82 (79.6) | Ref | Ref | Ref | Ref | Ref | Ref |  |
| Quartile 2 | 59 | 36 (61.0) | 0.77 (0.61, 0.96) | 0.75 (0.58, 0.97) | 0.75 (0.58, 0.97) | 0.83 (0.63, 1.10) | 0.75 (0.59, 0.97) | 0.83 (0.63, 1.11) |  |
| Quartiles 3 & 4 | 18 | 10 (55.6) | 0.70 (0.46, 1.07) | 0.84 (0.54, 1.33) | 0.84 (0.53, 1.32) | 0.98 (0.55, 1.73) | 0.77 (0.49, 1.22) | 0.99 (0.61, 1.61) |  |
| Caregiver’s highest level of education ^a^ | | | | | | | | | |
| None/primary | 103 | 80 (77.7) | Ref | Ref | Ref | Ref | Ref | Ref |  |
| Secondary | 50 | 35 (70.0) | 0.90 (0.73, 1.11) | 1.00 (0.77, 1.30) | 1.01 (0.78, 1.30) | 0.80 (0.58, 1.10) | 1.05 (0.83, 1.33) | 0.99 (0.75, 1.30) |  |
| College/Certificate | 27 | 13 (48.2) | 0.62 (0.41, 0.93) | 0.79 (0.51, 1.24) | 0.79 (0.51, 1.24) | 0.67 (0.41, 1.11) | 0.77 (0.51, 1.18) | 0.71 (0.40, 1.27) |  |
| Age (years) | | | | | | | | | |
| 0-9 | 42 | 32 (76.2) | Ref | Ref | Ref | Ref | Ref | Ref |  |
| 10-14 | 62 | 48 (77.4) | 1.02 (0.82, 1.26) | 0.98 (0.77, 1.25) | 0.98 (0.77, 1.25) | 1.02 (0.74, 1.41) | 1.02 (0.82, 1.27) | 1.09 (0.83, 1.44) |  |
| ≥15 | 78 | 49 (62.8) | 0.82 (0.65, 1.05) | 0.75 (0.57, 1.00) | 0.76 (0.57, 1.00) | 0.83 (0.59, 1.17) | 0.83 (0.62, 1.10) | 0.91 (0.66, 1.25) |  |
| Sex | | | | | | | | | |
| Male | 92 | 66 (71.7) | Ref | - | - | - | - | - |  |
| Female | 90 | 63 (70.0) | 0.98 (0.81, 1.18) | - | - | - | - | - |  |
| Parent’s vital status | | | | | | | | | |
| Both alive | 130 | 95 (73.1) | Ref | - | - | - | - | - |  |
| One parent died | 40 | 27 (67.5) | 0.92 (0.73, 1.17) | - | - | - | - | - |  |
| Both died | 12 | 7 (58.3) | 0.80 (0.49, 1.30) | - | - | - | - | - |  |
| **Nutritional, immunologic, virologic and treatment characteristics** | | | | | | | | | |
| Underweight / thin ^c^ | | | | | | | | | |
| No | 126 | 90 (71.4) | Ref | - | - | - | Ref | - |  |
| Yes | 21 | 17 (81.0) | 1.13 (0.90, 1.43) | - | - | - | 1.04 (0.82, 1.32) | - |  |
| CD4+ T-cell (cells/mm^3^) ^d^ | | | | | | | | | |
| ≥500 | 99 | 73 (73.7) | Ref | - | - | - | - | Ref |  |
| <500 | 17 | 11 (64.7) | 0.88 (0.61, 1.27) | - | - | - | - | 0.97 (0.68, 1.37) |  |
| HIV viral load (copies/mL) ^e^ | | | | | | | | | |
| ≥50 | 29 | 22 (75.9) | 1.11 (0.87, 1.40) | 1.11 (0.88, 1.41) | - | - | - | - |  |
| <50 | 118 | 81 (68.6) | Ref | Ref | - | - | - | - |  |
| HIV viral load (copies/mL) ^e^ | | | | | | | | | |
| ≥1000 | 9 | 7 (77.8) | 1.12 (0.78, 1.61) | - | 0.95 (0.66, 1.39) | - | - | - |  |
| <1000 | 138 | 96 (69.6) | Ref | - | Ref | - | - | - |  |
| Adherence ^f^ | | | | | | | | | |
| >95% | 91 | 60 (63.9) | Ref | - | - | Ref | - | - |  |
| ≤95% | 35 | 35 (71.4) | 1.08 (0.84, 1.40) | - | - | 1.11 (0.86, 1.44) | - | - |  |
| 3TC: lamivudine; ABC: abacavir; ART: antiretroviral therapy; AZT: zidovudine; DTG: dolutegravir; EFV: efavirenz; FTC: emtricitabine; LPVR: lopinovir/ritonavir; TAF: tenofovir alafenamide; TDF: tenofovir disoproxil fumarate  ^a^ Measured at enrollment into the cohort; all other characteristics measured at study visit where HFIAS and HWISE modules were administered [median = 10.2 years (25th, 75th percentiles: 6.9, 12.0) after enrollment]  ^b^ Based on socioeconomic status scale derived from data on housing characteristics, drinking water source, energy used for cooking, and asset ownership [20]; grouped into quartiles corresponding to scores of 0-6 (quartile 1; lowest), 7-12 (quartile 2), 13-18 (quartile 3), and 19-24 (quartile 4; highest)  ^c^ Underweight defined by weight-for-age Z score <-2 for children <5 years of age; thin defined by body mass index-for-age Z score <-2 for children and adolescents ≥5 years of age  ^d^ CD4 only available for 116 participants (63.7%); closest measure within 12 months prior the study visit was used [median = 182 days (25th, 75th percentiles: 0, 266) from measurement to study visit]  ^e^ HIV viral load only available for 147 participants (80.8%); closest measure within 12 months prior the study visit was used [median = 84 days (25th, 75th percentiles: 0, 182) from measurement to study visit]  ^f^ Adherence only available for 126 participants (69.2%); measured by pill count and defined based on the lowest measure for all drugs counted at the study visit | | | | | | | | | |
